# The role and mechanisms of myokines in sarcopenia: new intervention strategies for the challenges of aging

**DOI:** 10.3389/fmed.2025.1665708

**Published:** 2025-10-09

**Authors:** Xiangran Cui, Hongfei Liu, Yantong Liu, Zhitong Yu, Deyu Wang, Wei Wei, Shixuan Wang

**Affiliations:** Department of Orthopedics, Second Affiliated Hospital of Liaoning University of Traditional Chinese Medicine, Shenyang, China

**Keywords:** sarcopenia, muscle metabolism, myokines, muscle aging, therapeutic interventions

## Abstract

Sarcopenia is a major health issue among the global aging population, with a prevalence of 10 to 30% in those over 60 years old. As age advances, the gradual decline in muscle mass and function leads to reduced ability to perform daily activities and significantly increases the risks of falls, fractures, disability, and mortality. Recent studies have shown that skeletal muscle is not only a locomotive organ but also an important endocrine organ that affects systemic metabolism by secreting a series of bioactive molecules known as myokines. The secretion patterns of myokines undergo significant changes during aging and the progression of sarcopenia. Protective factors such as IL-15 and IGF-1 decrease, while pathological factors like myostatin and Activin A increase. This imbalance subsequently leads to the continued decline in muscle mass and function, reflected in multiple mechanisms including disruption of protein synthesis and degradation, mitochondrial dysfunction, and chronic inflammatory states. This article systematically reviews the role of myokines in sarcopenia, clarifies their molecular mechanisms, and explores clinical application prospects, aiming to provide a theoretical basis and new intervention targets for the prevention and treatment of sarcopenia. Future research should focus on the dynamic changes, interactions, and targeted intervention strategies of myokines to address the challenges of global aging and improve the quality of life for the elderly population.

## 1 Introduction

With the accelerating aging of the global population, sarcopenia has become a significant issue affecting the health of elderly individuals. According to the latest research data, the prevalence of sarcopenia in people aged 60 and above is between 10 and 30%, and it can exceed 50% in those over 80 years old (1–3). It is predicted that by 2050, the prevalence of sarcopenia among the global population aged 60 and above will reach 20%, continuing to increase in older age groups (4). Sarcopenia not only leads to a decline in the ability to perform daily activities but also significantly raises the risks of falls, fractures, disability, and mortality. Studies show that sarcopenia increases the risk of falls by 2 to 3 times and all-cause mortality by 1.5 to 2.5 times, placing a heavy burden on individuals, families, and society (5, 6). Data from the United States indicate that annual healthcare costs related to sarcopenia exceed $18.5 billion (7), representing a major public health economic burden (8, 9).

The traditional view holds that skeletal muscle is merely an organ of movement, but recent research has revealed that skeletal muscle is also an important endocrine organ capable of secreting a variety of bioactive molecules known as myokines (10–12). This breakthrough concept originated from the pioneering discovery by the Danish scholar Bente Klarlund Pedersen and her team in 2000. They observed that during exercise, the contraction of skeletal muscle produces and releases interleukin-6 (IL-6), and that this release is independent of immune cells, coming directly from the muscle fibers themselves (13, 14). This finding marked a paradigm shift in the understanding of skeletal muscle function and established the important concept of myokines. Subsequent studies have shown that these factors regulate not only the growth and metabolism of the muscle itself through autocrine, paracrine, and endocrine mechanisms but also participate in the regulation of multiple systems throughout the body, including adipose tissue, liver, skeleton, immune system, and nervous system (12, 15, 16).

The pathogenesis of sarcopenia is highly complex, involving multilayered and multifactorial pathophysiological changes (17). At the molecular level, the disruption of the balance between muscle protein synthesis and degradation is the central mechanism, with excessive activation of the ubiquitin-proteasome system (UPS) and the autophagy-lysosome pathway leading to increased net muscle protein degradation. Meanwhile, abnormal activation of the myostatin/activin A-Smad2/3 signaling pathway and inhibition of the IGF-1/PI3K/Akt/mTOR pathway further exacerbate the imbalance by reducing protein synthesis and increasing degradation (18, 19). Additionally, mitochondrial dysfunction, increased oxidative stress, and chronic low-grade inflammation (“inflammaging”) form a mutually reinforcing vicious cycle, resulting in the accumulation of reactive oxygen species (ROS), activation of NF-κB, and elevated release of pro-inflammatory cytokines (such as IL-6 and TNF-*α*) (20). These changes not only directly promote the degradation of myofibrillar proteins but also impair satellite cell function and muscle regenerative capacity.

Sarcopenia involves a complex disorder of the neuro-muscular-endocrine interaction network. Reduced neural innervation and motor unit remodeling lead to preferential loss of fast-twitch muscle fibers (type II), while impairment of neuromuscular junction integrity accelerates muscle denervation (21). Endocrinologically, declines in sex hormone levels, impaired insulin/IGF-1 signaling, and vitamin D deficiency collectively contribute to the progression of sarcopenia (22, 23). Notably, recent studies have revealed a key role of dysregulated “organ crosstalk” between adipose tissue and skeletal muscle in the pathogenesis of sarcopenia; for example, adipokines secreted by adipose tissue such as RBP4 can directly interfere with muscle metabolism and function (24). Additionally, gut microbiota dysbiosis, by affecting short-chain fatty acid production, inflammation levels, and nutrient absorption, is also considered an emerging mechanism in sarcopenia development. These multi-system, multi-level pathological changes interact with each other, ultimately leading to progressive declines in skeletal muscle mass and function (25, 26).

During the occurrence and progression of sarcopenia, the secretion patterns of myokines undergo significant changes, becoming important mediators of disease development (27–29). Specifically, the secretion of protective myokines (such as irisin and IL-15) decreases, while the secretion of pathogenic myokines (such as myostatin and activin A) increases. This imbalance leads to a progressive decline in muscle mass and function by affecting multiple pathways, including the balance of protein synthesis and degradation, mitochondrial function, and satellite cell activity (30).

**Figure 1:**
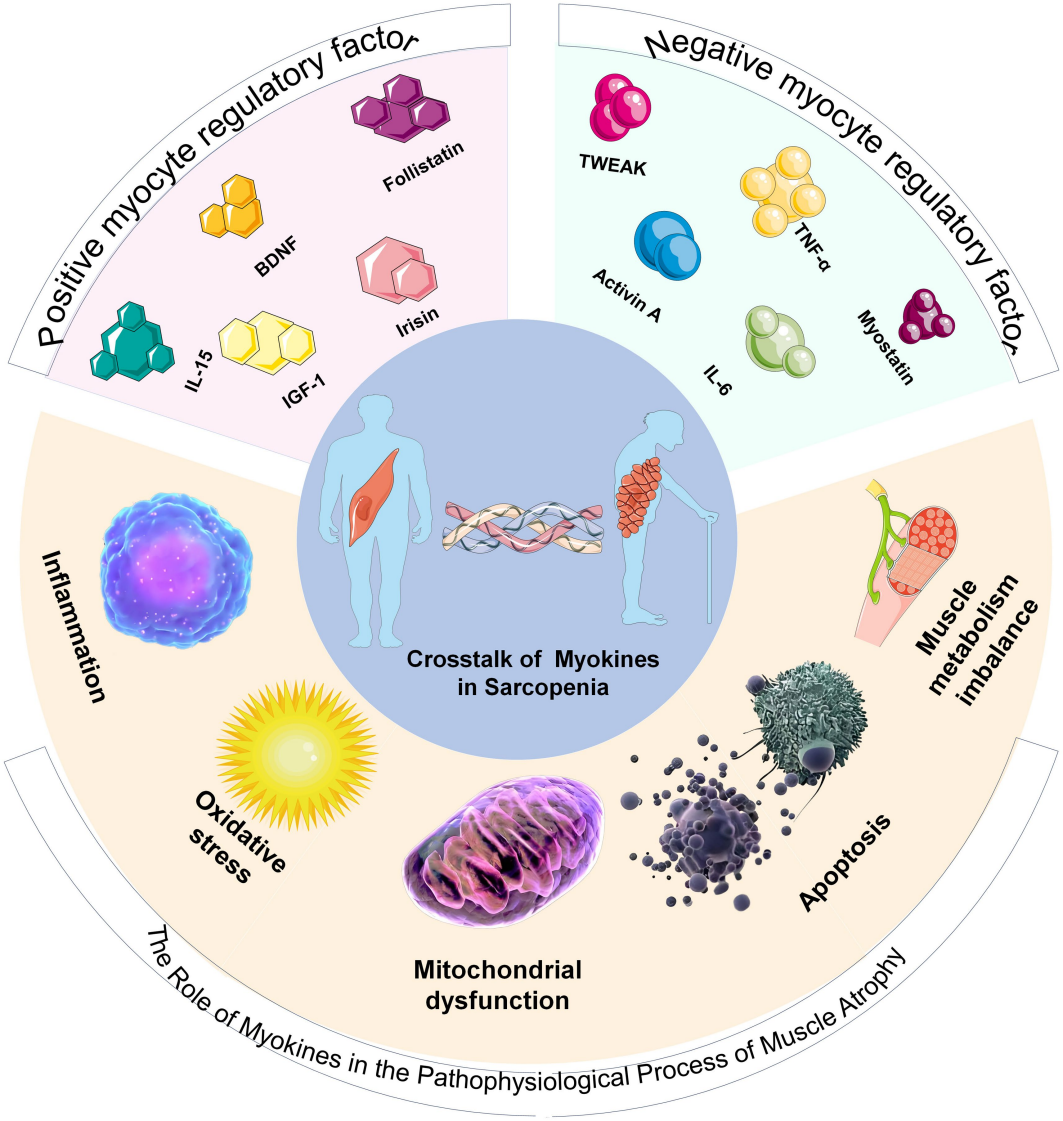
Schematic diagram of the classification of myokines and their relationship with sarcopenia. The upper part of the pie chart displays positive and negative myokine regulators. The lower part illustrates the pathophysiological process by which myokines lead to the development of sarcopenia.

This article aims to systematically review the role of myokines in sarcopenia, clarify their molecular mechanisms, explore clinical application prospects, and provide a theoretical foundation and new intervention targets for the prevention and treatment of the disease (Figure 1).

## 2 Biological functions of Myokines

**Figure 2:**
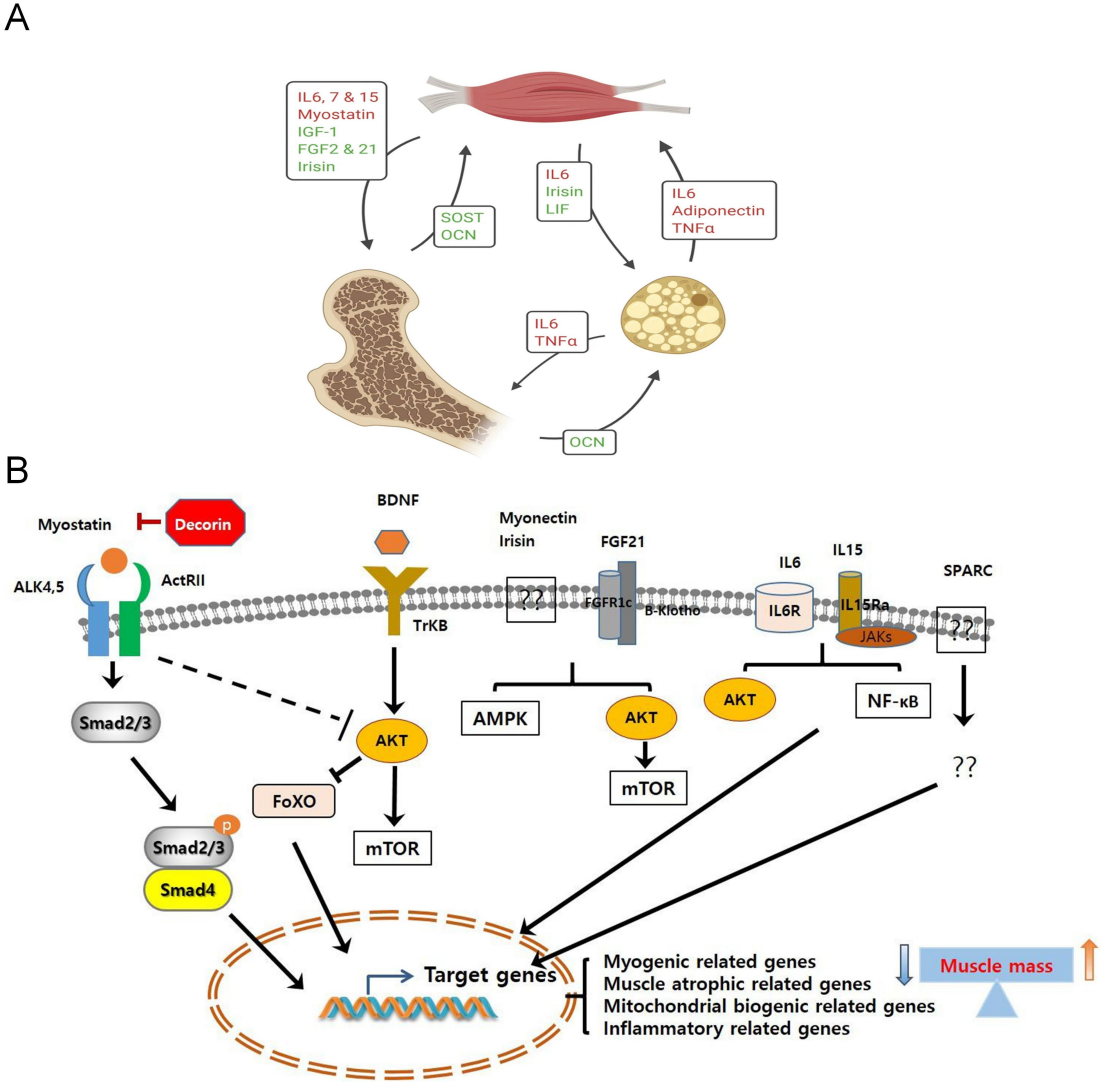
**(A)** The biological roles of myokines, osteokines, and adipokines in the interactions among muscle, bone, and fat (156). Copyright 2020, SPRINGER. **(B)** The signaling pathways of myokines induced by muscle contraction. The signaling pathways mediated by myokines lead to the expression of their target genes, thereby regulating muscle cell proliferation, differentiation, and growth. Ultimately, this results in an increase or decrease in muscle mass (30). Copyright 2019, FRONTIERS MEDIA SA.

Myokines are a large group of peptides, small proteins, and hormone-like molecules synthesized and secreted by skeletal muscle fibers in response to contraction or stress stimuli (Figures 2A,B). They exert their effects through autocrine, paracrine, and long-range endocrine mechanisms (10, 11, 30, 31). Since IL-6 was first defined as a myokine in 2003, researchers have identified more than 600 potential members, covering classic cytokines (IL-6, IL-8, IL-15) (32), growth factors (IGF-1, FGF21), hormone-like proteins (Irisin, Apelin) (33), chemokines (CXCL1), and protease inhibitors, among others (34). The discovery of myokines has greatly enriched understanding of the interactions between muscles and whole-body metabolism, inflammation regulation, and energy homeostasis. Skeletal muscle is thus regarded as a dynamic endocrine organ that (35), The discovery of myokines has greatly enriched understanding of the interactions between muscles and whole-body metabolism, inflammation regulation, and energy homeostasis. Skeletal muscle is thus regarded as a dynamic endocrine organ that, by secreting these bioactive substances, enables “cross-organ communication” with other organs and tissues such as the liver, adipose tissue, brain, heart, and even the immune system (15, 36–39), regulating systemic energy balance, inflammation levels, and organ function (40, 41). Myokines are generally divided into two categories: positive regulatory factors and negative regulatory factors. Positive regulatory myokines act as a “protective network” against muscle atrophy, with common functions summarized as promoting synthesis, enhancing regeneration, protecting energy (42), and combating inflammation (43), playing a vital biological role in maintaining and promoting skeletal muscle health. These factors actively prevent muscle wasting by enhancing protein synthesis, activating muscle satellite cell proliferation and differentiation, and supporting the structural integrity of neuromuscular junctions (44, 45). Conversely, the network of negative myokines reflects a vicious cycle of inhibiting synthesis, promoting breakdown, impairing regeneration (46), and amplifying inflammation (47), which mainly drives declines in muscle mass and strength by suppressing protein synthesis, encouraging protein degradation, and damaging repair mechanisms (48, 49).

### 2.1 Positive myocyte regulatory factor

#### 2.1.1 Il-15

IL-15 is an important myokine with significant anti-sarcopenic effects (50). It primarily maintains muscle mass by promoting protein synthesis and inhibiting protein degradation (51). It primarily maintains muscle mass by promoting protein synthesis and inhibiting protein degradation. Studies have shown that IL-15 can reduce the activation of protein degradation pathways and promote the activation and proliferation of muscle satellite cells, thereby enhancing the repair and regeneration capacity of muscle fibers. Additionally, IL-15 can reduce the accumulation of adipose tissue and improve intramuscular fat infiltration, which is beneficial for preventing and delaying sarcopenia caused by aging or disease (52, 53). After exercise, the secretion of IL-15 increases, making it one of the key molecular mechanisms through which exercise combats sarcopenia.

IL-15, as a key molecular hub regulating muscle metabolism, exhibits fascinating complexity in its functional regulation. Under physiological conditions, IL-15 maintains the dynamic balance of muscle tissue by finely modulating muscle stem cell fate, promoting protein synthesis, and mitochondrial biogenesis. However, in aging and pathological microenvironments, the molecular mechanisms of IL-15 undergo profound shifts: epigenetic modifications, inflammatory signaling networks, and genetic polymorphisms together weave a dynamic regulatory molecular landscape (54). Specific genetic variations may lead to dysfunction in the IL-15 signaling pathway, causing it to transform from a muscle-protective factor into a potential inducer of inflammation and muscle degeneration (55). This molecular fate reversal not only reveals the complex pathogenesis of sarcopenia but also provides new targets and theoretical foundations for precision medicine, embodying the profound concept of “function as destiny” in molecular biology research.

#### 2.1.2 IGF-1

IGF-1 is a key regulator of muscle metabolism, maintaining muscle mass by promoting protein synthesis and activating satellite cells (56, 57). With aging and the progression of chronic disease, IGF-1 expression and activity decline markedly, a change closely associated with the development of sarcopenia (58). IGF-1 can antagonize negative regulators such as myostatin and plays an important role in maintaining muscle homeostasis. Therefore, increasing IGF-1 levels is considered a potential strategy for preventing and treating sarcopenia.

As a major molecular regulator of muscle metabolism, IGF-1 precisely controls the proliferation and differentiation of muscle stem cells under physiological conditions, preserving the structural integrity of muscle tissue. However, in the microenvironment of aging and chronic disease, IGF-1’s function can undergo significant changes influenced by epigenetic modifications and inflammatory networks (59). This reversal of molecular fate reveals the complex pathogenesis of sarcopenia and provides new targets and a theoretical basis for precision medicine.

#### 2.1.3 Irisin

Irisin is a myokine released in response to exercise, derived from the cleavage of fibronectin type III domain-containing protein 5 (FNDC5) in skeletal muscle (60). Its anti-sarcopenic effects are mainly reflected in promoting mitochondrial biogenesis and function, enhancing muscle energy metabolism and endurance (61). Additionally, irisin can induce the “browning” of white adipose tissue, increasing energy expenditure and reducing ectopic fat deposition in muscles (38, 62). Studies have found that irisin also activates muscle satellite cells, facilitating muscle regeneration, and optimizes systemic metabolic conditions by improving insulin sensitivity (63, 64). Irisin levels tend to be reduced in older adults and patients with sarcopenia, while exercise training can effectively increase its secretion; this is one of the mechanisms by which exercise improves sarcopenia.

As a newly discovered myokine, irisin plays an important role in regulating energy metabolism and muscle health, with complex and diverse mechanisms. In healthy individuals, irisin supports muscle growth and function by promoting caloric utilization in muscle cells and conversion of adipose tissue. However, during the development of sarcopenia, irisin’s actions are subtly regulated by various environmental and genetic factors. Environmental factors such as exercise volume, nutritional status, and inflammatory responses significantly affect irisin release and activity. For example, moderate exercise can upregulate irisin secretion and enhance its protective effects on muscle (65). Under conditions of chronic inflammation or metabolic syndrome, irisin’s action may be suppressed, leading to energy imbalance and exacerbated muscle degeneration (66). In addition, genetic variations may cause interindividual differences in irisin levels, revealing the complexity of its biological regulation. This molecular diversity not only provides deeper insight into the pathology of sarcopenia but also lays a foundation for potential therapeutic strategies.

#### 2.1.4 BDNF

BDNF is not only a neurotrophic factor but also an important myokine secreted by skeletal muscle. In the defensive mechanism against sarcopenia, BDNF mainly ensures effective neural control over muscles by maintaining the integrity and function of the neuromuscular junction (67). It promotes neuron survival and axonal growth, enhances synaptic transmission efficiency, and prevents age-related degeneration of neuromuscular connections. Additionally, BDNF is involved in regulating mitochondrial biogenesis and oxidative metabolism in muscles, improving muscle fatigue resistance and metabolic health (68, 69). Research shows that exercise can significantly increase the expression and secretion of BDNF in muscles, which is an important molecular basis for the protective effects of exercise on the neuromuscular system (70, 71).

Under healthy conditions, BDNF not only promotes the survival and development of nerve cells but also supports the maintenance of muscle quality by regulating the proliferation and differentiation of muscle stem cells. However, during the progression of sarcopenia, BDNF function is profoundly influenced by environmental and genetic factors (72). For example, a sedentary lifestyle and chronic inflammatory states suppress BDNF expression, reducing its bioactivity in muscle and further exacerbating muscle atrophy. In contrast, regular exercise can significantly elevate BDNF levels, activate downstream signaling pathways, and improve muscle health (73). Additionally, an individual’s genetic background affects the expression and function of the BDNF gene; specific single nucleotide polymorphisms may lead to notable changes in BDNF activity, resulting in varying sarcopenia risks among different populations. The interplay of these mechanisms reveals the multiple roles of BDNF in muscle metabolism and neuromuscular adaptation, providing new research perspectives for the treatment of sarcopenia.

#### 2.1.5 FGF21

FGF21 is a factor expressed in muscle and liver that plays multiple important roles in combating sarcopenia. It can improve insulin sensitivity, optimize glucose and lipid metabolism, and reduce fat infiltration and inflammation in muscle (74, 75). FGF21 also enhances mitochondrial function (76), increases energy utilization efficiency, and mitigates oxidative stress-induced damage to muscle fibers (77). During exercise, FGF21 secretion increases, which is one of the mechanisms by which exercise protects muscle quality through regulating the network of myokines.

Generally, FGF21 regulates metabolism and energy balance by promoting fat oxidation and inhibiting muscle atrophy to maintain muscle mass (78). Under healthy conditions, moderate exercise and proper nutrition can significantly increase FGF21 levels, enhancing its biological effects. However, in environments characterized by chronic inflammation, obesity, or aging, the function of FGF21 is suppressed, which may lead to weakened muscle synthesis (79). Additionally, genetic polymorphisms also affect the expression and activity of FGF21, resulting in individual differences in muscle adaptation to metabolic stress. This interaction between environmental and genetic factors reveals the potential value of FGF21 in the prevention and treatment of sarcopenia, providing possible avenues for personalized interventions.

#### 2.1.6 Follistatin

Follistatin, as a natural antagonist of myostatin and Activin A, promotes muscle growth and maintenance by binding to and neutralizing the activities of these negative regulatory factors. It blocks the binding of myostatin to its receptor, thereby lifting the inhibition on muscle growth and promoting the activation of muscle satellite cells and muscle fiber hypertrophy (80, 81). Studies have shown that increased expression of follistatin can significantly enhance muscle repair and regeneration capacity, slowing the progression of sarcopenia (82, 83). During exercise, follistatin expression is upregulated, which is one of the key mechanisms through which resistance training promotes muscle hypertrophy. Therefore, enhancing the expression or activity of follistatin is considered a promising strategy for the treatment of sarcopenia (84).

In healthy individuals, moderate exercise and proper nutrition can upregulate the expression of follistatin, enhancing muscle synthesis capacity. However, environmental factors such as chronic inflammation and obesity can interfere with follistatin’s function, leading to reduced activity and promoting muscle imbalance (85). At the same time, polymorphisms in the genetic background may affect the expression level of the follistatin gene, resulting in significant differences among individuals. This interaction between environmental and genetic factors reveals the potential value of follistatin in the treatment of sarcopenia, providing new perspectives for the application of personalized medicine.

### 2.2 Negative myocyte regulatory factor

#### 2.2.1 Myostatin

Myostatin is the most important negative myokine, primarily synthesized and secreted by skeletal muscle cells, specifically inhibiting muscle growth and development. At the molecular level, myostatin binds to Activin receptor type II (ActRII) (86), activating the Smad2/3 signaling pathway, which suppresses the activation and proliferation of muscle satellite cells while promoting the degradation of muscle fiber proteins (87). Studies have shown that myostatin levels tend to be elevated in patients with sarcopenia (88). In conditions of chronic disease, aging, and prolonged inactivity, increased myostatin expression is a key molecular mechanism underlying muscle atrophy (89, 90).

As a potent inhibitor of muscle growth, myostatin leads to muscle atrophy by suppressing the proliferation and differentiation of muscle cells. In healthy individuals, moderate exercise and good nutrition can effectively reduce myostatin levels, promoting muscle synthesis. However, specific genetic variations can cause abnormalities in myostatin function, which are observed in certain families and manifest as significantly enhanced muscle development. Environmental factors such as chronic inflammation and metabolic disorders can activate the myostatin pathway, exacerbating the progression of sarcopenia (91). Understanding the dual role of myostatin in both healthy and pathological states not only provides potential targets for the treatment of sarcopenia but also lays the foundation for developing personalized intervention strategies.

#### 2.2.2 Activin a

Activin A and myostatin belong to the same TGF-*β* superfamily and are both important negative regulators of muscle. Activin A functions through a mechanism similar to myostatin, binding to the ActRII receptor which inhibits the activation of muscle satellite cells and the synthesis of muscle fiber proteins. In various chronic disease-associated muscle-wasting conditions, such as cancer cachexia and chronic kidney disease, Activin A levels are significantly elevated (92, 93). Studies have shown that Activin A not only acts directly on muscle but also affects systemic metabolic states, such as promoting inflammation in adipose tissue and insulin resistance, further exacerbating the progression of muscle wasting (94). Therefore, targeting the Activin A signaling pathway has become an important therapeutic strategy for treating muscle wasting (95).

It promotes muscle atrophy by enhancing protein degradation pathways and inhibiting synthesis pathways in muscle cells. In healthy individuals, Activin A levels can decrease with exercise and nutritional status, facilitating muscle growth. However, genetic factors such as single nucleotide polymorphisms may affect the expression of Activin A, leading to individual differences in muscle adaptability. Meanwhile, environmental factors like inflammation, obesity, or metabolic disorders can upregulate Activin A levels, worsening the condition of sarcopenia (96). This indicates that the balance between promoting and inhibiting muscle synthesis by Activin A is regulated by complex environmental and genetic factors, providing new insights for the precision treatment of sarcopenia.

#### 2.2.3 Il-6

IL-6 plays a dual role in sarcopenia, depending on its expression level, duration, and physiological context (97). During acute exercise, IL-6 transiently increases as a “myokine,” promoting glucose uptake, fat oxidation, and anti-inflammatory effects. However, in chronic inflammatory states (such as in the elderly or cancer patients), persistently elevated IL-6 exerts pro-inflammatory and muscle-wasting effects. Chronic high levels of IL-6 activate the JAK/STAT3 signaling pathway, upregulating the expression of muscle atrophy-related ubiquitin ligases like MuRF1 and Atrogin-1, thereby increasing protein degradation (98, 99). Additionally, IL-6 can induce oxidative stress and mitochondrial dysfunction, further accelerating muscle fiber damage and atrophy (100).

Typically, elevated IL-6 levels in inflammatory environments activate muscle degradation pathways, leading to the loss of muscle tissue. However, during moderate exercise and acute pathological states, IL-6 helps facilitate muscle regeneration and supports the repair process. Genetic factors, such as IL-6 gene polymorphisms, influence an individual’s inflammatory response, resulting in differences in muscle adaptability and sarcopenia risk. Environmental factors like chronic inflammation and aging may promote sustained high expression of IL-6, exacerbating muscle decline (101). This complex regulatory relationship positions IL-6 as an emerging therapeutic target for sarcopenia, highlighting the importance of personalized interventions.

#### 2.2.4 TNF-*α*

TNF-α is a major pro-inflammatory cytokine that plays an important role in various chronic disease-associated sarcopenia (102). It promotes muscle atrophy through multiple mechanisms: first, TNF-*α* activates the NF-κB signaling pathway, upregulating the expression of atrophy-related genes such as MuRF1 and Atrogin-1, thereby increasing protein ubiquitination and degradation, second, it inhibits the expression of myogenic transcription factors like MyoD, weakening muscle regeneration capacity, third, TNF-*α* promotes oxidative stress and mitochondrial dysfunction, leading to increased apoptosis (26). Studies have also found that TNF-α can enhance the expression and activity of myostatin, producing a synergistic pro-atrophy effect (103). Elevated TNF-*α* levels are significantly correlated with decreased muscle mass and function in inflammatory diseases, cancer cachexia, and age-related sarcopenia.

In an inflammatory microenvironment, TNF-α can significantly activate the ubiquitin-proteasome pathway, accelerating muscle protein degradation and promoting muscle atrophy. However, under healthy conditions, moderate exercise and nutritional interventions can effectively regulate TNF-*α* levels and maintain muscle homeostasis. Genetic factors, such as TNF-α gene polymorphisms, lead to individual differences in sensitivity to inflammatory responses, affecting the progression rate of muscle loss. Environmental factors like chronic diseases, obesity, and aging can persistently upregulate TNF-α expression, exacerbating muscle degeneration (104). This complex environment-genetic interaction reveals the multidimensional role of TNF-α in the regulation of muscle metabolism and provides a new theoretical basis for personalized interventions.

#### 2.2.5 Tweak

TWEAK is a member of the TNF superfamily that activates multiple signaling pathways by binding to the Fn14 receptorpromoting muscle atrophy (105). At the molecular level, TWEAK inhibits satellite cell activation and differentiation, weakening muscle regeneration capacity; simultaneously, it promotes the activation of the ubiquitin-proteasome system and the autophagy-lysosome system, increasing protein degradation (106, 107). Studies have shown that the activation of the TWEAK/Fn14 signaling pathway is closely associated with muscle atrophy in various chronic disease models. In addition, TWEAK can induce muscle cells to produce inflammatory factors, forming a positive feedback loop that further exacerbates muscle damage and atrophy (108, 109). Therefore, targeting the TWEAK/Fn14 signaling pathway is considered a potential strategy for treating sarcopenia.

Under pathological conditions, TWEAK activates the Fn14 receptor, promoting the initiation of muscle atrophy pathways and significantly accelerating the degeneration of muscle tissue. However, in healthy individuals, moderate expression of TWEAK can stimulate the activation of muscle stem cells and the muscle regeneration process. Genetic factors such as variations in the TWEAK gene can affect its biological activity, leading to significant differences in individual muscle adaptability. Environmental factors including chronic inflammation, metabolic disorders, and aging can substantially regulate TWEAK expression levels, further complicating its role in muscle metabolism (110). This multilayered regulatory mechanism reveals the potential therapeutic value of TWEAK in sarcopenia, offering new perspectives for precision medicine research.

## 3 The role of myokines in muscle atrophy and cytokine changes in different types of sarcopenia

### 3.1 The role of myokines in the pathophysiological process of muscle atrophy

#### 3.1.1 Inflammation

**Figure 3:**
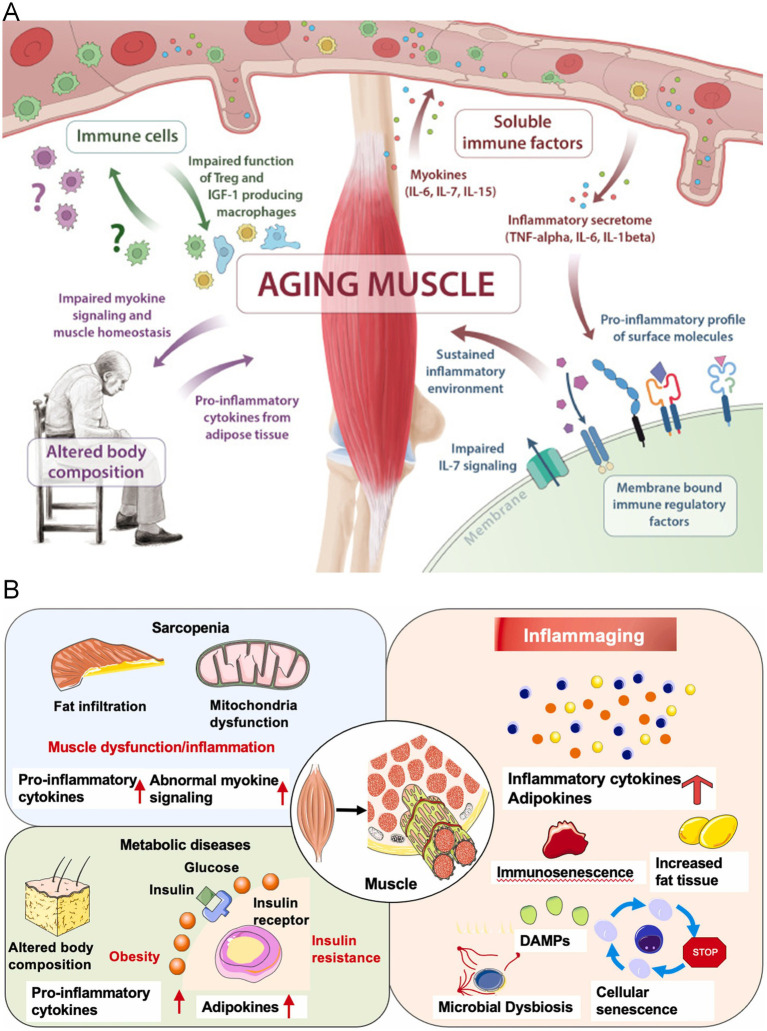
**(A)** The release of myokines from skeletal muscle aging is central to the pathogenesis of immunosenescence and sarcopenia. Multiple pathways are affected, including insufficient myokine signaling (IL-6, IL-7, IL-15), the shift of membrane-bound immune regulatory factors toward pro-inflammatory features, impaired immune cell function, and altered body composition (157). Copyright 2019 ELSEVIER. **(B)** The link between inflammation development and sarcopenia. Under aging and risk factor conditions, the sustained and increased levels of inflammatory cytokines, adipokines, and myokines in the serum may make muscles more susceptible to sarcopenia (158). Copyright 2022 ELSEVIER IRELAND LTD.

In sarcopenia, pro-inflammatory myokines such as IL-6 and TNF-*α* are persistently overexpressed, activating the JAK/STAT and NF-κB signaling pathways, which induce and maintain a chronic low-grade inflammatory state (111, 112). This not only directly promotes the expression of protein degradation markers like MuRF1 and Atrogin-1 but also recruits immune cells such as macrophages to infiltrate, exacerbating inflammation in the muscle fiber microenvironment (113). At the same time, chronic inflammation impairs the self-renewal and differentiation potential of muscle satellite cells, weakening muscle regeneration and repair. Additionally, the relative decrease in anti-inflammatory myokines (such as IL-10) makes it more difficult to reverse the inflammation (114) (Figure 3).

Against a background of chronic inflammation, the spleen, as a central immune organ, plays a key regulatory role in the development of sarcopenia (68). Dynamic changes in the spleen’s immune cell repertoire—particularly decreased NK cell activity and reduced B cell diversity—directly affect the metabolic balance of muscle tissue. By secreting inflammatory factors (such as TNF-*α* and IL-6) and modulating cytokine networks, the spleen not only amplifies inflammatory signals but also interferes with muscle protein synthesis and the regenerative capacity of muscle stem cells (115). In cancer-associated sarcopenia, immune remodeling of the spleen is especially pronounced; its exosomes and intercellular communication mechanisms further accelerate muscle tissue degeneration, creating a negative feedback loop between inflammation and muscle metabolism (116). This complex mechanism reveals the immune system’s deeper pathological regulatory role in muscle loss.

#### 3.1.2 Oxidative stress

Patients with sarcopenia experience a decline in the activity of antioxidant pathways such as Nrf2, leading to sustained elevated oxidative stress. Muscle cytokines like TNF-*α* and IL-1β can further activate NADPH oxidase and upregulate the production of reactive oxygen species (ROS) (117, 118). This not only causes oxidative damage to lipids, proteins, and DNA but also promotes the activation of inflammatory and apoptotic signaling pathways such as NF-κB and p38 MAPK (119), creating a “inflammation-oxidative stress” positive feedback loop that drives the progression of muscle atrophy (120, 121). Some positive factors, such as Irisin and FGF21, alleviate oxidative damage by enhancing antioxidant enzyme expression and mitochondrial homeostasis.

#### 3.1.3 Mitochondrial dysfunction

Abnormal regulation of myocyte cytokines significantly affects mitochondrial structure and energy function. Under chronic conditions of elevated IL-6 and TNF-*α*, excessive mitophagy, loss of membrane potential, and impaired energy production are prominent, worsening muscle endurance and repair capacity (122, 123). Conversely, factors such as Irisin, BDNF, and FGF21 can promote mitochondrial biogenesis by upregulating pathways like PGC-1α, maintaining or restoring mitochondrial quantity and function, improving the metabolic environment, and thereby partially offsetting motor dysfunction and fatigue (124, 125).

#### 3.1.4 Apoptosis

Pro-inflammatory myogenic factors (such as TNF-α and TWEAK) can upregulate the caspase cascade in muscle cells and activate the mitochondria-mediated apoptosis pathway, accelerating the loss of muscle fibers and satellite cells (126, 127). Anti-apoptotic myogenic factors (such as IGF-1 and IL-15) inhibit autophagy and apoptosis processes, protecting the structural integrity of muscle. Apoptosis driven jointly by inflammatory factors and oxidative stress is a key reason for the rapid decline in muscle fiber number in sarcopenia (128).

#### 3.1.5 Imbalance between muscle protein synthesis and degradation

Myokines play a central role in regulating muscle protein metabolism. Factors such as IGF-1, IL-15, and Irisin activate the PI3K/Akt/mTOR pathway, promoting protein synthesis (129, 130). In contrast, Myostatin, Activin A, and high concentrations of IL-6 and TNF-*α* activate Smad2/3 and FOXO signaling, upregulating ubiquitin-proteasome system-related enzymes (such as MuRF1 and Atrogin-1), leading to accelerated protein degradation. The outcome is impaired protein synthesis and enhanced degradation, resulting in irreversible net muscle loss, which represents the most direct molecular basis of sarcopenia (131, 132).

### 3.2 Age-related sarcopenia

The myokine network in age-related sarcopenia exhibits characteristic changes (133). Firstly, the pro-inflammatory myokine system is systemically upregulated, manifested by a persistent mild increase in circulating levels of TNF-*α*, IL-6, and IL-1β, constituting the so-called “inflammaging” phenomenon. Studies have shown that individuals over 80 years old have serum IL-6 levels on average 2 to 4 times higher than young adults, which is significantly negatively correlated with muscle mass and function decline (134, 135). Secondly, myostatin expression and activity are significantly increased; multiple studies have confirmed that its serum levels rise with age and positively correlate with decreased muscle strength. Meanwhile, protective myokines such as IGF-1, BDNF, and irisin are secreted less, especially local muscle-derived IGF-1, whose expression is markedly reduced in elderly muscle. In addition, the myokine response to exercise stimulation in older muscles is blunted, characterized by reduced peak secretion and shortened duration of pro-myogenic factors like IL-15 and follistatin after exercise, while negative regulators such as myostatin are insufficiently suppressed. This “muscle endocrine aging” leads to a diminished responsiveness of muscle protein synthesis to nutritional and exercise stimuli and is a crucial mechanism underlying age-related sarcopenia (136).

### 3.3 Disease-related sarcopenia

#### 3.3.1 Cancer cachexia

Cytokine changes in muscle cells are the most severe and specific in cancer cachexia. The most notable feature is the large synergistic increase of various pro-inflammatory and pro-catabolic factors, including TNF-*α* (also known as cachectin), IL-6, IL-1β, TWEAK, and Activin A. Studies have shown that serum Activin A levels in advanced cancer patients can be elevated 5 to 10 times compared to healthy controls and are closely associated with the rate of muscle loss (137). Additionally, local expression of MIC-1/GDF15 in skeletal muscle of cancer patients is significantly increased, promoting appetite suppression and protein breakdown (138). Notably, certain tumors (such as pancreatic cancer) also secrete specific factors like PTHrP, which further exacerbate muscle atrophy. At the same time, protective myocytic factors such as IGF-1 and IL-15 are downregulated, and the muscle response to exercise-induced myogenic IL-6 in cancer patients shifts from anti-inflammatory to pro-inflammatory characteristics. Furthermore, recent research has found a significant increase in the ratio of Myostatin to Follistatin in the muscles of cancer cachexia patients, disrupting the balance of this key antagonistic pair. These changes make cancer cachexia the most rapidly progressing and difficult-to-treat form of muscle wasting (139).

In elderly patients, cancer-induced sarcopenia is particularly severe, and its pathogenic mechanisms go far beyond those of ordinary aging, presenting a compounded “multiple-hit” effect: first, aging itself has already reduced the regenerative capacity of muscle stem cells and the efficiency of protein synthesis, while cancer, by activating complex inflammatory cascades and metabolic reprogramming, precisely “targets” the muscle tissue’s self-repair mechanisms, driving muscle cells into irreversible catabolism and apoptosis (140, 141). Large amounts of proinflammatory factors in the tumor microenvironment (such as TNF-*α* and IL-6) not only disrupt muscle protein synthesis pathways but also suppress mitochondrial energy metabolism and stem cell activity, creating a self-reinforcing vicious cycle that leads to rapid, irreversible muscle loss in elderly patients. This distinctive pathological process makes cancer the most aggressive driver of muscle wasting in older adults—essentially a biological-level “accelerated aging” mechanism (142).

#### 3.3.2 Sarcopenia associated with CKD

In chronic kidney disease-related sarcopenia, changes in myokines are closely associated with the accumulation of uremic toxins. Firstly, the uremic environment stimulates skeletal muscle to produce a large amount of pro-inflammatory myokines, particularly IL-6 and TNF-*α*, resulting in sustained activation of both local and systemic inflammation (143, 144). Studies have shown that NF-κB activity in the muscle tissue of dialysis patients is significantly elevated, further amplifying the inflammatory response. Secondly, the myostatin/Activin signaling pathway is overactivated in the muscles of CKD patients, accompanied by increased levels of skeletal muscle and circulating Activin A, which positively correlates with the degree of renal function decline. At the same time, the IGF-1/Akt/mTOR pathway is suppressed, partly due to uremic toxins interfering with IGF-1 receptor sensitivity. Additionally, skeletal muscle expression of FGF23 is increased while Klotho expression is decreased in CKD patients, disrupting this critical axis and accelerating muscle protein degradation (145). Notably, renal anemia and secondary hyperparathyroidism further exacerbate abnormalities in these myokines, creating a vicious cycle. The secretion response of muscle-derived IL-15 and irisin after exercise in dialysis patients is significantly lower than in healthy controls, which may be one reason why exercise benefits are limited in this population (146, 147).

#### 3.3.3 Sarcopenia associated with chronic heart failure

The cytokine profile changes in muscle cells of patients with chronic heart failure (CHF) reflect the effects of low perfusion and sympathetic nervous system activation (148). Firstly, levels of circulating and local muscle TNF-*α* and IL-6 in CHF patients are significantly elevated and positively correlated with the severity of cardiac dysfunction. Studies show that serum TNF-α in NYHA class III-IV patients is on average 2–3 times higher than in healthy controls. Secondly, BDNF expression in skeletal muscle of heart failure patients is reduced, while Myostatin expression is increased; the imbalance between these two leads to impaired neuromuscular junction integrity and decreased muscle protein synthesis. Meanwhile, due to reduced cardiac output and insufficient tissue oxygenation, the expression of aerobic metabolism-related myokines such as FGF21 and Irisin is decreased in the muscles of CHF patients, exacerbating mitochondrial dysfunction (149). Notably, CHF patients exhibit abnormal myokine responses post-exercise, characterized by delayed clearance of IL-6 and insufficient secretion of protective factors. Furthermore, research has found that *β*-adrenergic blocker treatment can partially improve the disturbed myokine profile in CHF patients, which may be one mechanism by which prognosis is improved (150). Overall, the changes in myokines in CHF patients lie between those seen in age-related sarcopenia and cancer cachexia but exhibit distinctive cardiogenic characteristics.

#### 3.3.4 Disuse muscle atrophy

In disuse muscle atrophy, changes in muscle cell cytokines are mainly driven by mechanical unloading and reduced activity. First, bed rest, plaster immobilization, or microgravity environments rapidly upregulate myostatin expression. Studies have shown that short-term (7 days) bed rest can increase local muscle Myostatin mRNA expression by 2–3 times. Meanwhile, IGF-1 expression significantly decreases under disuse conditions, with a more pronounced decline in fast-twitch fibers (type II) compared to slow-twitch fibers (type I). Second, under disuse conditions, the secretion of neurotrophic muscle cytokines such as BDNF and NT-4 decreases, leading to impaired neuromuscular junction integrity and accelerated muscle denervation (151). Additionally, IL-15 expression decreases in microgravity and inactivity, while IL-6 and TNF-*α* show slight increases, though this inflammatory response is generally less severe than in other types of muscle wasting (152). Notably, changes in muscle cell cytokines during disuse muscle atrophy are characterized by rapidity and reversibility. The serum Follistatin/Myostatin ratio decreases in long-term bedridden patients but quickly recovers after resuming activity. These features make disuse muscle atrophy the most responsive type of muscle wasting to exercise interventions (153). Recent studies have found that the lack of mechanical stimulation suppresses the YAP/TAZ signaling pathway in muscle cells, further exacerbating the upregulation of negative muscle cell cytokines, providing new targets for targeted interventions.

### 3.4 Mechanisms of Myokines in the treatment of sarcopenia

The mechanisms by which myokines act in the treatment of sarcopenia involve multiple complex biological pathways. At the molecular level, myokines such as irisin, IL-6, and BDNF directly regulate muscle metabolism and regeneration by activating key intracellular signaling pathways. Specifically, irisin can markedly enhance mitochondrial biogenesis by activating the PGC-1α and AMPK pathways, improving myocyte energy metabolism efficiency, it also modulates the browning of adipose tissue, indirectly improving metabolic status. IL-6, through the JAK–STAT and mTOR signaling pathways, promotes satellite cell activation and differentiation, enhances muscle protein synthesis, and suppresses the ubiquitin–proteasome degradation pathways associated with muscle atrophy. These myokines are not merely passive signaling molecules but active regulators of muscle metabolism, resistance to oxidative stress, and inflammation. More importantly, they can mediate interorgan communication and modulate immune system function, potentially reversing age-related muscle degeneration and offering novel molecular targets and intervention strategies for treating sarcopenia.

Recent research advances show that CRISPR-based interventions have potential as innovative treatments for sarcopenia (154). For example, some studies have successfully edited the myostatin gene using the CRISPR/Cas9 system, significantly increasing muscle mass in small livestock such as pigs. Another study investigated precise CRISPR editing of the myostatin signaling pathway in skeletal muscle, demonstrating positive effects on improving muscle quality and function. In addition, research has reported generating myostatin-mutant rabbits through gene editing, a result that provides new directions for personalized interventions (155). These advances not only open new potential avenues for treating sarcopenia but also underscore the key role of CRISPR in improving muscle health.

## 4 Conclusion and discussion

We employed a systematic literature search strategy to conduct a comprehensive and precise review in the Web of Science and PubMed database. During the search process, we used multiple carefully selected keyword combinations, including “myokines,” “muscle cytokines,” “sarcopenia,” and “age-related muscle atrophy,” setting the time frame from 2013 to 2023 to comprehensively capture the latest research developments in this field. To ensure the representativeness and quality of the literature, we prioritized human clinical studies and animal experimental models and conducted a rigorous quality assessment of the included studies, evaluating the scientific rigor of the study design, sample size, the sophistication of experimental methods, and the reliability of conclusions. Through this strict search and screening approach, we aim to provide a more systematic, objective, and cutting-edge literature review.

Current research on myokines in sarcopenia faces numerous limitations. First, most studies are based on animal models or *in vitro* experiments, posing challenges for clinical translation. Second, there is a wide variety of myokines, and the interactions among different myokines as well as their combined effects within the human body have not been fully elucidated. Additionally, current research predominantly focuses on several major myokines (such as IL-6, IL-15, and myostatin), while functional studies on other novel myokines remain relatively insufficient. At the same time, although a large body of research has examined the roles of IL-6, IGF-1, and myostatin in sarcopenia, there remains some controversy regarding the mechanisms by which these factors act. For example, IL-6 displays bidirectional effects depending on the context: during acute inflammation it can promote muscle repair, whereas in chronic inflammation it may lead to muscle wasting. This suggests that IL-6’s function depends not only on its concentration but also on specific physiological conditions. In addition, while IGF-1 supplementation therapy can improve muscle quality in the short term, long-term use may encounter issues of reduced organismal responsiveness, indicating that treatment regimens should be chosen cautiously in clinical practice. Furthermore, the function of myostatin is not limited to inhibiting muscle growth; some studies suggest it may also indirectly affect muscle health by influencing insulin signaling pathways and fat metabolism. Therefore, future research should comprehensively consider the interactions among these factors to elucidate the complexity of sarcopenia and to advance the development of personalized intervention strategies.

Future research directions should emphasize the development of longitudinal cohort studies to clarify the dynamic expression patterns of myokines with aging and their association with sarcopenia progression. At the same time, in-depth investigation into the interaction networks between myokines and other tissue stromal factors is necessary, especially their cross-talk with adipose tissue and the skeletal system. Another important direction is to elucidate the regulatory mechanisms by which different types of exercise modulate the expression of specific myokines, in order to optimize exercise intervention strategies.

At the forefront of sarcopenia research, the integration of novel myogenic factor discoveries with CRISPR gene editing technology offers revolutionary possibilities for precise intervention. By constructing multi-scale, cross-dimensional regulatory network models of myogenic factors, we can deeply analyze the molecular mechanisms of muscle degeneration. Specifically, this innovative research paradigm goes beyond traditional single-factor studies by adopting a systems biology perspective that integrates complex regulatory patterns involving epigenetic regulation, intercellular communication networks, and inflammatory responses. For example, precise editing of key genes associated with muscle atrophy using CRISPR-Cas9 technology, combined with single-cell transcriptomics and proteomics analyses, can reveal the multi-level mechanisms by which myogenic factors regulate muscle metabolism, stem cell activity, and inflammatory responses. This multidimensional integrative approach is expected to overcome the limitations of conventional research paradigms, providing a more in-depth and comprehensive theoretical foundation for personalized and precise interventions in sarcopenia, while also opening new research avenues for muscle health management in the elderly population.

Myokines, as bioactive molecules secreted by muscle tissue, play a complex and crucial role in the onset and progression of sarcopenia. Through various mechanisms—including regulating the balance of muscle protein metabolism, influencing the activation and differentiation of muscle satellite cells, participating in the modulation of inflammatory responses, and maintaining the integrity of neuromuscular junctions—myokines serve as an important bridge connecting mechanical stimuli, metabolic changes, and muscle adaptive responses. Among them, key myokines such as IL-6, IL-15, BDNF, myostatin, and decorin have irreplaceable roles in maintaining muscle mass and function, and their dysregulated expression directly contributes to the pathological process of sarcopenia.

Intervention strategies targeting myokines provide new perspectives and approaches for the prevention and treatment of sarcopenia. On one hand, exercise training, especially resistance training, can optimize the secretion profile of myokines by enhancing the expression of muscle growth-promoting factors and inhibiting the production of muscle atrophy-inducing factors. On the other hand, pharmacological treatments targeting specific myokines—such as anti-myostatin antibodies and delivery of exogenous IL-15 or irisin—have demonstrated potential in preventing muscle atrophy in animal studies, with some interventions currently advancing toward clinical trial phases.

With the continuous deepening of our understanding of the biological functions of myokines and the ongoing development of precise intervention technologies, personalized treatment strategies based on myokines are expected to become important means to address the global challenge of aging and improve the quality of life in elderly populations. In the fields of geriatric and rehabilitation medicine, research on myokines will provide a strong theoretical foundation and practical guidance for improving sarcopenia-related functional impairments and preventing disability, ultimately contributing to the realization of healthy aging.

## References

[1] Chen C Liao D-M. Sarcopenia: a review. Tungs Med J. (2024) 18:S23–7. doi: 10.4103/etmj.etmj-d-24-00008

[2] Scisciola L Fontanella RA Surina S Cataldo V Paolisso G Barbieri M. Sarcopenia and cognitive function: role of myokines in muscle brain cross-talk. Life-Basel. (2021) 11:173. doi: 10.3390/life11020173, PMID: 33672427 PMC7926334

[3] Tagliafico AS Bignotti B Torri L Rossi F. Sarcopenia: how to measure, when and why. Radiol Med. (2022) 127:228–37. doi: 10.1007/s11547-022-01450-3, PMID: 35041137 PMC8960583

[4] Shao M Wang Q Lv Q Zhang Y Gao G Lu S. Advances in the research on myokine-driven regulation of bone metabolism. Heliyon. (2024) 10:e22547. doi: 10.1016/j.heliyon.2023.e22547, PMID: 38226270 PMC10788812

[5] Damluji AA Alfaraidhy M AlHajri N Rohant NN Kumar M Al Malouf C. Sarcopenia and cardiovascular diseases. Circulation. (2023) 147:1534–53. doi: 10.1161/circulationaha.123.064071, PMID: 37186680 PMC10180053

[6] Larsson L Degens H Li M Salviati L Lee YI Thompson W. Sarcopenia: aging-related loss of muscle mass and function. Physiol Rev. (2019) 99:427–511. doi: 10.1152/physrev.00061.2017, PMID: 30427277 PMC6442923

[7] Janssen I Shepard DS Katzmarzyk PT Roubenoff R. The healthcare costs of sarcopenia in the United States. J Am Geriatr Soc. (2004) 52:80–5. doi: 10.1111/j.1532-5415.2004.52014.x, PMID: 14687319

[8] Beaudart C Rizzoli R Bruyere O Reginster J-Y Biver E. Sarcopenia: burden and challenges for public health. Arch Public Health. (2014) 72:45–5. doi: 10.1186/2049-3258-72-45, PMID: 25810912 PMC4373245

[9] Bruyere O Beaudart C Ethgen O Reginster J-Y Locquet M. The health economics burden of sarcopenia: a systematic review. Maturitas. (2019) 119:61–9. doi: 10.1016/j.maturitas.2018.11.003, PMID: 30502752

[10] Giudice J Taylor JM. Muscle as a paracrine and endocrine organ. Curr Opin Pharmacol. (2017) 34:49–55. doi: 10.1016/j.coph.2017.05.005, PMID: 28605657 PMC5808999

[11] Schnyder S Handschin C. Skeletal muscle as an endocrine organ: PGC-1α, myokines and exercise. Bone. (2015) 80:115–25. doi: 10.1016/j.bone.2015.02.008, PMID: 26453501 PMC4657151

[12] Severinsen MCK Pedersen BK. Muscle-organ crosstalk: the emerging roles of Myokines. Endocr Rev. (2020) 41:594–609. doi: 10.1210/endrev/bnaa016, PMID: 32393961 PMC7288608

[13] Pedersen BK Febbraio MA. Muscle as an endocrine organ: focus on muscle-derived interleukin-6. Physiol Rev. (2008) 88:1379–406. doi: 10.1152/physrev.90100.2007, PMID: 18923185

[14] Pedersen BK. From the discovery of myokines to exercise as medicine. Dan Med J. (2023) 70:766. PMID: 37622635

[15] Hoffmann C Weigert C. Skeletal muscle as an endocrine organ: the role of Myokines in exercise adaptations. Cold Spring Harb Perspect Med. (2017) 7:a029793. doi: 10.1101/cshperspect.a029793, PMID: 28389517 PMC5666622

[16] Leal LG Lopes MA Batista ML. Physical exercise-induced Myokines and muscle-adipose tissue crosstalk: a review of current knowledge and the implications for health and metabolic diseases. Front Physiol. (2018) 9:1307. doi: 10.3389/fphys.2018.01307, PMID: 30319436 PMC6166321

[17] Mercuri E Bönnemann CG Muntoni F. Muscular dystrophies. Lancet. (2019) 394:2025–38. doi: 10.1016/S0140-6736(19)32910-1, PMID: 31789220

[18] Foltz SJ Luan J Call JA Patel A Peissig KB Fortunato MJ. Four-week rapamycin treatment improves muscular dystrophy in a fukutin-deficient mouse model of dystroglycanopathy. Skelet Muscle. (2016) 6:20. doi: 10.1186/s13395-016-0091-9, PMID: 27257474 PMC4890530

[19] Rico A Guembelzu G Palomo V Martinez A Aiastui A Casas-Fraile L. Allosteric modulation of GSK-3β as a new therapeutic approach in limb girdle muscular dystrophy R1 calpain 3-related. Int J Mol Sci. (2021) 22:7367. doi: 10.3390/ijms22147367, PMID: 34298987 PMC8308041

[20] Millozzi F Papait A Bouché M Parolini O Palacios D. Nano-immunomodulation: a new strategy for skeletal muscle diseases and aging? Int J Mol Sci. (2023) 24:1175. doi: 10.3390/ijms24021175, PMID: 36674691 PMC9862642

[21] Cruz-Jentoft AJ Bahat G Bauer J Boirie Y Bruyère O Cederholm T. Sarcopenia: revised European consensus on definition and diagnosis. Age Ageing. (2019) 48:16–31. doi: 10.1093/ageing/afy169, PMID: 30312372 PMC6322506

[22] Nishikawa H Asai A Fukunishi S Nishiguchi S Higuchi K. Metabolic syndrome and sarcopenia. Nutrients. (2021) 13:3519. doi: 10.3390/nu13103519, PMID: 34684520 PMC8541622

[23] Wilson D Jackson T Sapey E Lord JM. Frailty and sarcopenia: the potential role of an aged immune system. Ageing Res Rev. (2017) 36:1–10. doi: 10.1016/j.arr.2017.01.006, PMID: 28223244

[24] Li C-w Yu K Shyh-Chang N Jiang Z Liu T Ma S. Pathogenesis of sarcopenia and the relationship with fat mass: descriptive review. J Cachexia Sarcopenia Muscle. (2022) 13:781–94. doi: 10.1002/jcsm.12901, PMID: 35106971 PMC8977978

[25] Chen H-T Chung Y-C Chen Y-J Ho S-Y Wu H-J. Effects of different types of exercise on body composition, muscle strength, and IGF-1 in the elderly with Sarcopenic obesity. J Am Geriatr Soc. (2017) 65:827–32. doi: 10.1111/jgs.14722, PMID: 28205203

[26] Thoma A Lightfoot AP. NF-kB and inflammatory cytokine Signalling: role in skeletal muscle atrophy. Adv Exp Med Biol. (2018) 2018:267–79. doi: 10.1007/978-981-13-1435-3_12, PMID: 30390256

[27] Bernasconi P Carboni N Ricci G Siciliano G Politano L Maggi L. Elevated TGF β2 serum levels in Emery-Dreifuss muscular dystrophy: implications for myocyte and tenocyte differentiation and fibrogenic processes. Nucleus. (2018) 9:292–304. doi: 10.1080/19491034.2018.1467722, PMID: 29693488 PMC5973167

[28] Iwata Y Katanosaka Y Arai Y Komamura K Miyatake K Shigekawa M. A novel mechanism of myocyte degeneration involving the Ca2+−permeable growth factor-regulated channel. J Cell Biol. (2003) 161:957–67. doi: 10.1083/jcb.200301101, PMID: 12796481 PMC2172975

[29] Ujihara Y Kanagawa M Mohri S Takatsu S Kobayashi K Toda T. Elimination of fukutin reveals cellular and molecular pathomechanisms in muscular dystrophy-associated heart failure. Nat Commun. (2019) 10:5754. doi: 10.1038/s41467-019-13623-2, PMID: 31848331 PMC6917736

[30] Lee JH Jun H-S. Role of Myokines in regulating skeletal muscle mass and function. Front Physiol. (2019) 10:42. doi: 10.3389/fphys.2019.00042, PMID: 30761018 PMC6363662

[31] Kapilevich LV Kabachkova AV Zakharova AN Lalaeva GS Kironenko TA Dyakova EY. Secretory function of skeletal muscles: producing mechanisms and myokines physiological effects. Usp Fiziol Nauk. (2016) 47:7–26. 27530041

[32] Ciaraldi TP Ryan AJ Mudaliar SR Henry RR. Altered Myokine secretion is an intrinsic property of skeletal muscle in type 2 diabetes. PLoS One. (2016) 11:e0158209. doi: 10.1371/journal.pone.0158209, PMID: 27453994 PMC4959771

[33] Besse-Patin A Montastier E Vinel C Castan-Laurell I Louche K Dray C. Effect of endurance training on skeletal muscle myokine expression in obese men: identification of apelin as a novel myokine. Int J Obes. (2014) 38:707–13. doi: 10.1038/ijo.2013.158, PMID: 23979219

[34] Lorincz H Somodi S Ratku B Harangi M Paragh G. Crucial regulatory role of Organokines in relation to metabolic changes in non-diabetic obesity. Meta. (2023) 13:270. doi: 10.3390/metabo13020270, PMID: 36837889 PMC9967669

[35] Karsenty G Olson EN. Bone and muscle endocrine functions: unexpected paradigms of inter-organ communication. Cell. (2016) 164:1248–56. doi: 10.1016/j.cell.2016.02.043, PMID: 26967290 PMC4797632

[36] Suzuki S Yamanouchi K Soeta C Katakai Y Harada R Naito K. Skeletal muscle injury induces hepatocyte growth factor expression in spleen. Biochem Biophys Res Commun. (2002) 292:709–14. doi: 10.1006/bbrc.2002.6706, PMID: 11922624

[37] Eckel J. Myokines in metabolic homeostasis and diabetes. Diabetologia. (2019) 62:1523–8. doi: 10.1007/s00125-019-4927-9, PMID: 31263909

[38] Li F Li Y Duan Y Hu C-AA Tang Y Yin Y. Myokines and adipokines: involvement in the crosstalk between skeletal muscle and adipose tissue. Cytokine Growth Factor Rev. (2017) 33:73–82. doi: 10.1016/j.cytogfr.2016.10.003, PMID: 27765498

[39] Saponaro F Bertolini A Baragatti R Galfo L Chiellini G Saba A. Myokines and microbiota: new perspectives in the endocrine muscle-gut axis. Nutrients. (2024) 16:4032. doi: 10.3390/nu16234032, PMID: 39683426 PMC11643575

[40] Iizuka K Machida T Hirafuji M. Skeletal muscle is an endocrine organ. J Pharmacol Sci. (2014) 125:125–31. doi: 10.1254/jphs.14R02CP, PMID: 24859778

[41] Pedersen BK. Muscle as a secretory organ. Compr Physiol. (2013) 3:1337–62. doi: 10.1002/j.2040-4603.2013.tb00522.x, PMID: 23897689

[42] Zhang L Lv JJ Wang CY Ren YY Yong M. Myokine, a key cytokine for physical exercise to alleviate sarcopenic obesity. Mol Biol Rep. (2023) 50:2723–34. doi: 10.1007/s11033-022-07821-3, PMID: 36571655

[43] Piccirillo R. Exercise-induced myokines with therapeutic potential for muscle wasting. Front Physiol. (2019) 10:287. doi: 10.3389/fphys.2019.00287, PMID: 30984014 PMC6449478

[44] Albrecht E Schering L Liu Y Komolka K Kuehn C Wimmers K. Triennial growth and development symposium: factors influencing bovine intramuscular adipose tissue development and cellularity. J Anim Sci. (2017) 95:2244–54. doi: 10.2527/jas.2016.1036, PMID: 28726981

[45] Senesi P Luzi L Terruzzi I. Adipokines, Myokines, and Cardiokines: the role of nutritional interventions. Int J Mol Sci. (2020) 21:372. doi: 10.3390/ijms21218372, PMID: 33171610 PMC7664629

[46] Meacci E Chirco A Garcia-Gil M. Potential vitamin E signaling mediators in skeletal muscle. Antioxidants. (2024) 13:383. doi: 10.3390/antiox13111383, PMID: 39594525 PMC11591548

[47] Huang QR Wu ML Wu XY Zhang YW Xia Y. Muscle-to-tumor crosstalk: the effect of exercise-induced myokine on cancer progression. Biochim Biophys Acta. (2022) 1877:188761. doi: 10.1016/j.bbcan.2022.188761, PMID: 35850277

[48] Liu Z Ye X Li N Shang J Du G Yang X. Roles of natural products on myokine expression and secretion in skeletal muscle atrophy. Gen Comp Endocrinol. (2024) 355:114550. doi: 10.1016/j.ygcen.2024.114550, PMID: 38768928

[49] Mancinelli R Checcaglini F Coscia F Gigliotti P Fulle S Fano-Illic G. Biological aspects of selected Myokines in skeletal muscle: focus on aging. Int J Mol Sci. (2021) 22:520. doi: 10.3390/ijms22168520, PMID: 34445222 PMC8395159

[50] Lenert ME Szabo-Pardi TA Burton MD. Regulatory T-cells and IL-5 mediate pain outcomes in a preclinical model of chronic muscle pain. Mol Pain. (2023) 19:691. doi: 10.1177/17448069221110691, PMID: 35712872 PMC9926397

[51] Huan X Zhao R Song J Zhong HH Su M Yan C. Increased serum IL-2, IL-4, IL-5 and IL-12p70 levels in AChR subtype generalized myasthenia gravis. BMC Immunol. (2022) 23:26. doi: 10.1186/s12865-022-00501-8, PMID: 35624411 PMC9145157

[52] Merriwether EN Agalave NM Dailey DL Rakel BA Kolker SJ Lenert ME. IL-5 mediates monocyte phenotype and pain outcomes in fibromyalgia. Pain. (2021) 162:1468–82. doi: 10.1097/j.pain.0000000000002089, PMID: 33003107 PMC7987864

[53] Barker T Rogers VE Henriksen VT Dixon BM Momberger NG Rasmussen GL. Muscular-based and patient-reported outcomes differentially associate with circulating superoxide dismutases and cytokines in knee osteoarthritis. Cytokine. (2019) 115:45–9. doi: 10.1016/j.cyto.2018.11.034, PMID: 30634097

[54] Antonaci S Polignano A Tortorella C Garofalo AR Jirillo E Bonomo L. Role of interleukin 2, interleukin 4 and interleukin 5 in the T helper cell-driven B cell polyclonal differentiation in the elderly. Cytobios. (1992) 70:77–85. PMID: 1451534

[55] Ren W Wang Z Wang J Wu Z Ren Q Yu A. IL-5 overexpression attenuates aortic dissection by reducing inflammation and smooth muscle cell apoptosis. Life Sci. (2020) 241:117144. doi: 10.1016/j.lfs.2019.117144, PMID: 31830482

[56] Gomez JP Goncalves C Pichon C Midoux P. Effect of IL-1β, TNF-α and IGF-1 on trans-endothelial passage of synthetic vectors through an in vitro vascular endothelial barrier of striated muscle. Gene Ther. (2017) 24:416–24. doi: 10.1038/gt.2017.40, PMID: 28504656

[57] Lu W Xiao W Xie W Fu X Pan L Jin H. The role of Osteokines in sarcopenia: therapeutic directions and application prospects. Front Cell Dev Biol. (2021) 9:374. doi: 10.3389/fcell.2021.735374, PMID: 34650980 PMC8505767

[58] Sharma B Dabur R. Role of pro-inflammatory cytokines in regulation of skeletal muscle metabolism: a systematic review. Curr Med Chem. (2020) 27:2161–88. doi: 10.2174/0929867326666181129095309, PMID: 30488792

[59] Zhao L-D Bie L-Y Hu L Zhu Z-H Meng X-H Cong L-L. IGF-1 induces cellular senescence in rat articular chondrocytes via Akt pathway activation. Exp Ther Med. (2020) 20:1. doi: 10.3892/etm.2020.9177, PMID: 32952639 PMC7480142

[60] Farshbaf MJ Alviña K. Multiple roles in neuroprotection for the exercise derived myokine Irisin. Front Aging Neurosci. (2021) 13:649929. doi: 10.3389/fnagi.2021.649929, PMID: 33935687 PMC8086837

[61] Seo DY Bae JH Kim TN Kwak HB Kha PT Han J. Exercise-induced circulating Irisin level is correlated with improved cardiac function in rats. Int J Environ Res Public Health. (2020) 17:863. doi: 10.3390/ijerph17113863, PMID: 32485990 PMC7313080

[62] Shaw A Toth BB Kiraly R Arianti R Csomos I Poliska S. Irisin stimulates the release of CXCL1 from differentiating human subcutaneous and deep-neck derived adipocytes via upregulation of NFκB pathway. Front Cell Dev Biol. (2021) 9:872. doi: 10.3389/fcell.2021.737872, PMID: 34708041 PMC8542801

[63] Dong J Dong Y Dong Y Chen F Mitch WE Zhang L. Inhibition of myostatin in mice improves insulin sensitivity via irisin-mediated cross talk between muscle and adipose tissues. Int J Obes. (2016) 40:434–42. doi: 10.1038/ijo.2015.200, PMID: 26435323 PMC4783239

[64] Slate-Romano JJ Yano N Zhao TC. Irisin reduces inflammatory signaling pathways in inflammation-mediated metabolic syndrome. Mol Cell Endocrinol. (2022) 552:111676. doi: 10.1016/j.mce.2022.111676, PMID: 35569582 PMC10084474

[65] Reza MM Sim CM Subramaniyam N Ge X Sharma M Kambadur R. Irisin treatment improves healing of dystrophic skeletal muscle. Oncotarget. (2017) 8:98553–66. doi: 10.18632/oncotarget.21636, PMID: 29228710 PMC5716750

[66] Oranger A Storlino G Dicarlo M Zerlotin R Pignataro P Sanesi L. Impact of 10-day bed rest on serum levels of irisin and markers of musculoskeletal metabolism. FASEB J. (2023) 37:e22668. doi: 10.1096/fj.202201005RR, PMID: 36475382 PMC13281841

[67] Pang BPS Iu ECY Hang M Chan WS Tse MCL Yeung CTY. Deficiency of muscle-generated brain-derived neurotrophic factor causes inflammatory myopathy through reactive oxygen species-mediated necroptosis and pyroptosis. Redox Biol. (2024) 78:103418. doi: 10.1016/j.redox.2024.103418, PMID: 39531828 PMC11602578

[68] Aby K Antony R Eichholz M Srinivasan R Li Y. Enhanced pro-BDNF-p75NTR pathway activity in denervated skeletal muscle. Life Sci. (2021) 286:120067. doi: 10.1016/j.lfs.2021.120067, PMID: 34678261 PMC8595791

[69] Verbickas V Kamandulis S Snieckus A Venckunas T Baranauskiene N Brazaitis M. Serum brain-derived neurotrophic factor and interleukin-6 response to high-volume mechanically demanding exercise. Muscle Nerve. (2018) 57:E46–51. doi: 10.1002/mus.25687, PMID: 28500647

[70] Dicarlo M Pignataro P Zerlotin R Suriano C Zecca C Dell'Abate MT. Short-term Irisin treatment enhanced neurotrophin expression differently in the hippocampus and the prefrontal cortex of young mice. Int J Mol Sci. (2023) 24:9111. doi: 10.3390/ijms24119111, PMID: 37298063 PMC10252658

[71] Freitas DA Rocha-Vieira E Soares BA Nonato LF Fonseca SR Martins JB. High intensity interval training modulates hippocampal oxidative stress, BDNF and inflammatory mediators in rats. Physiol Behav. (2018) 184:6–11. doi: 10.1016/j.physbeh.2017.10.027, PMID: 29111230

[72] Zhang J Zhu L Zhou J Yu Q Yang G Luo C. BDNF alleviates senescence and enhances osteogenic differentiation in bone marrow mesenchymal stem cells via the TrkB/PI3K/AKT pathway. Tissue Cell. (2025) 96:972. doi: 10.1016/j.tice.2025.102972, PMID: 40367890

[73] Brown AD Marko AD Marko DM Baranowski BJ Silvera S Finch MS. Brain-derived neurotrophic factor drives muscle adaptation similar to aerobic training in mice. FASEB J. (2025) 39:421. doi: 10.1096/fj.202402421R, PMID: 39853792 PMC11760663

[74] Slusher AL Whitehurst M Zoeller RF Mock JT Maharaj M Huang CJ. Attenuated fibroblast growth factor 21 response to acute aerobic exercise in obese individuals. Nutr Metab Cardiovasc Dis. (2015) 25:839–45. doi: 10.1016/j.numecd.2015.06.002, PMID: 26141939

[75] dos Santos ARD Zanuso BD Miola VFB Barbalho SM Bueno PCS Flato UAP. Adipokines, myokines, and hepatokines: crosstalk and metabolic repercussions. Int J Mol Sci. (2021) 22:639. doi: 10.3390/ijms22052639, PMID: 33807959 PMC7961600

[76] Jena J García-Peña LM Pereira RO. The roles of FGF21 and GDF15 in mediating the mitochondrial integrated stress response. Front Endocrinol. (2023) 14:530. doi: 10.3389/fendo.2023.1264530, PMID: 37818094 PMC10561105

[77] Kim C-S Joe Y Choi H-S Back SH Park JW Chung HT. Deficiency of fibroblast growth factor 21 aggravates obesity-induced atrophic responses in skeletal muscle. J Inflamm. (2019) 16:17. doi: 10.1186/s12950-019-0221-3, PMID: 31312114 PMC6611052

[78] Luo X Zhang H Cao X Yang D Yan Y Lu J. Endurance exercise-induced Fgf21 promotes skeletal muscle Fiber conversion through TGF-β1 and p38 MAPK signaling pathway. Int J Mol Sci. (2023) 24:401. doi: 10.3390/ijms241411401, PMID: 37511159 PMC10379449

[79] Li X Hong Y He H Jiang G You W Liang X. FGF21 mediates mesenchymal stem cell senescence via regulation of mitochondrial dynamics. Oxidative Med Cell Longev. (2019) 2019:4915149. doi: 10.1155/2019/4915149, PMID: 31178962 PMC6501200

[80] Domin R Dadej D Pytka M Zybek-Kocik A Ruchala M Guzik P. Effect of various exercise regimens on selected exercise-induced cytokines in healthy people. Int J Environ Res Public Health. (2021) 18:1261. doi: 10.3390/ijerph18031261, PMID: 33572495 PMC7908590

[81] Tsompanidis A Vafiadaki E Bluher S Kalozoumi G Sanoudou D Mantzoros CS. Ciliary neurotrophic factor upregulates follistatin and Pak1, causes overexpression of muscle differentiation related genes and downregulation of established atrophy mediators in skeletal muscle. Metabolism. (2016) 65:915–25. doi: 10.1016/j.metabol.2016.03.005, PMID: 27173470

[82] Qi C Song X Wang H Yan Y Liu B. The role of exercise-induced myokines in promoting angiogenesis. Front Physiol. (2022) 13:577. doi: 10.3389/fphys.2022.981577, PMID: 36091401 PMC9459110

[83] Zocoler de Sousa CA Renno Sierra AP Martinez Galan BS de Sousa Maciel JF Manoel R Barbeiro HV. Time course and role of exercise-induced cytokines in muscle damage and repair after a Marathon race. Front Physiol. (2021) 12:752144. doi: 10.3389/fphys.2021.752144, PMID: 34721075 PMC8554198

[84] Szabo MR Pipicz M Csont T Csonka C. Modulatory effect of Myokines on reactive oxygen species in ischemia/reperfusion. Int J Mol Sci. (2020) 21:382. doi: 10.3390/ijms21249382, PMID: 33317180 PMC7763329

[85] Anuja AK Bhadu D Naveen R Singh MK Rai MK Agarwal V. High serum myostatin level suggests accelerated muscle senescence in active idiopathic inflammatory myositis. Indian J Rheumatol. (2021) 16:284–9. doi: 10.4103/injr.injr_309_20

[86] Zhou X Wang JL Lu J Song Y Kwak KS Jiao Q. Reversal of Cancer Cachexia and muscle wasting by ActRIIB antagonism leads to prolonged survival. Cell. (2010) 142:531–43. doi: 10.1016/j.cell.2010.07.011, PMID: 20723755

[87] Schiaffino S Dyar KA Ciciliot S Blaauw B Sandri M. Mechanisms regulating skeletal muscle growth and atrophy. FEBS J. (2013) 280:4294–314. doi: 10.1111/febs.12253, PMID: 23517348

[88] Guo B Zhang Z-K Liang C Li J Liu J Lu A. Molecular communication from skeletal muscle to bone: a review for muscle-derived Myokines regulating bone metabolism. Calcif Tissue Int. (2017) 100:184–92. doi: 10.1007/s00223-016-0209-4, PMID: 27830278

[89] Kawaguchi Y Watanabe A Shiratori T Kaku R Ueda K Okamoto K. Myostatin expression in lung cancer induces sarcopenia and promotes cancer progression. Gen Thorac Cardiovasc Surg. (2024) 72:232–9. doi: 10.1007/s11748-023-01969-w, PMID: 37648959

[90] Watanabe H Enoki Y Maruyama T. Sarcopenia in chronic kidney disease: factors, mechanisms, and therapeutic interventions. Biol Pharm Bull. (2019) 42:1437–45. doi: 10.1248/bpb.b19-00513, PMID: 31474705

[91] Jespersen J Kjaer M Schjerling P. The possible role of myostatin in skeletal muscle atrophy and cachexia. Scand J Med Sci Sports. (2006) 16:74–82. doi: 10.1111/j.1600-0838.2005.00498.x, PMID: 16533345

[92] Daitoku N Miyamoto Y Hiyoshi Y Tokunaga R Sakamoto Y Sawayama H. Activin a promotes cell proliferation, invasion and migration and predicts poor prognosis in patients with colorectal cancer. Oncol Rep. (2022) 47:318. doi: 10.3892/or.2022.8318, PMID: 35445735 PMC9073419

[93] de Ruiter RDD Wisse LEE Schoenmaker T Yaqub M Sanchez-Duffhues G Eekhoff EMW. TGF-beta induces activin a production in dermal fibroblasts derived from patients with fibrodysplasia ossificans progressiva. Int J Mol Sci. (2023) 24:2299. doi: 10.3390/ijms24032299, PMID: 36768622 PMC9916423

[94] McKee A Morley JE Matsumoto AM Vinik A. Sarcopenia: an endocrine disorder? Endocr Pract. (2017) 23:1140–9. doi: 10.4158/EP171795.RA, PMID: 28704095

[95] Martin A Gallot YS Freyssenet D. Molecular mechanisms of cancer cachexia-related loss of skeletal muscle mass: data analysis from preclinical and clinical studies. J Cachexia Sarcopenia Muscle. (2023) 14:1150–67. doi: 10.1002/jcsm.13073, PMID: 36864755 PMC10235899

[96] Wang H Zhang Q Kaplan FS Pignolo RJ. Clearance of senescent cells from injured muscle abrogates heterotopic ossification in mouse models of Fibrodysplasia Ossificans Progressiva. J Bone Miner Res. (2022) 37:95–107. doi: 10.1002/jbmr.4458, PMID: 34633114 PMC8770661

[97] Lightfoot AP Cooper RG. The role of myokines in muscle health and disease. Curr Opin Rheumatol. (2016) 28:661–6. doi: 10.1097/BOR.0000000000000337, PMID: 27548653

[98] Hardee JP Fix DK Wang X Goldsmith FC Koh H-J Carson JA. Systemic IL-6 regulation of eccentric contraction-induced muscle protein synthesis. Am J Phys Cell Phys. (2018) 315:C91–C103. doi: 10.1152/ajpcell.00063.2018, PMID: 29641213 PMC6087730

[99] Gao S Durstine JL Koh H-J Carver WE Frizzell N Carson JA. Acute myotube protein synthesis regulation by IL-6-related cytokines. Am J Phys Cell Phys. (2017) 313:C487–500. doi: 10.1152/ajpcell.00112.2017, PMID: 28768641 PMC5792171

[100] Moresi V Adamo S Berghella L. The JAK/STAT pathway in skeletal muscle pathophysiology. Front Physiol. (2019) 10:500. doi: 10.3389/fphys.2019.00500, PMID: 31114509 PMC6502894

[101] Xiong L Zhao K Cao Y Guo H-H Pan J-X Yang X. Linking skeletal muscle aging with osteoporosis by Lamin a/C deficiency. PLoS Biol. (2020) 18:e3000731. doi: 10.1371/journal.pbio.3000731, PMID: 32479501 PMC7310860

[102] Zhao Q Yang ST Wang JJ Zhou J Xing SS Shen CC. TNF alpha inhibits myogenic differentiation of C2C12 cells through NF-κB activation and impairment of IGF-1 signaling pathway. Biochem Biophys Res Commun. (2015) 458:790–5. doi: 10.1016/j.bbrc.2015.02.026, PMID: 25686491

[103] Rodriguez J Fernández-Verdejo R Pierre N Priem F Francaux M. Endurance training attenuates catabolic signals induced by TNF-α in muscle of mice. Med Sci Sports Exerc. (2016) 48:227–34. doi: 10.1249/MSS.0000000000000756, PMID: 26285024

[104] Shirakami Y Kato J Maeda T Ideta T Imai K Sakai H. Skeletal muscle atrophy is exacerbated by steatotic and fibrotic liver-derived TNF-α in senescence-accelerated mice. J Gastroenterol Hepatol. (2023) 38:800–8. doi: 10.1111/jgh.16171, PMID: 36890117

[105] Yadav A Singh A Phogat J Dahuja A Dabur R. Magnoflorine prevent the skeletal muscle atrophy via Akt/mTOR/FoxO signal pathway and increase slow-MyHC production in streptozotocin-induced diabetic rats. J Ethnopharmacol. (2021) 267:113510. doi: 10.1016/j.jep.2020.113510, PMID: 33141056

[106] Yadav A Dabur R. Ursolic acid restores redox homeostasis and pro-inflammatory cytokine production in denervation-induced skeletal muscle atrophy. Appl Biochem Biotechnol. (2024) 197:1152–73. doi: 10.1007/s12010-024-05059-2, PMID: 39361198

[107] Dutt V Saini V Gupta P Kaur N Bala M Gujar R. S-allyl cysteine inhibits TNFα-induced skeletal muscle wasting through suppressing proteolysis and expression of inflammatory molecules. BBA-Gen Subjects. (2018) 1862:895–906. doi: 10.1016/j.bbagen.2017.12.015 29288771

[108] Yang MG Ge HZ Ji SQ Li Y Xu L Bi ZJ. TWEAK and Fn14 are overexpressed in immune-mediated necrotizing myopathy: implications for muscle damage and repair. Rheumatology. (2023) 62:3732–41. doi: 10.1093/rheumatology/kead108, PMID: 36916753

[109] Zhou J Liu B Liang C Li YX Song YH. Cytokine signaling in skeletal muscle wasting. Trends Endocrinol Metab. (2016) 27:335–47. doi: 10.1016/j.tem.2016.03.002, PMID: 27025788

[110] Wei C Liu X Miao Z Zhang H Wang Y Qi G. TWEAK/Fn14 axis may promote vascular smooth muscle cell senescence via p38 signaling pathway: preliminary evidence. Future Sci OA. (2025) 11:906. doi: 10.1080/20565623.2025.2455906, PMID: 39840833 PMC11756581

[111] Liang J-L Xie J-F Wang C-Y Chen N. Regulatory roles of micrornas in sarcopenia and exercise intervention. Sheng Li Xue Bao. (2020) 72:667–76. PMID: 33106837

[112] Zhou Y-Z Chen P-J Xiao W-H. Mechanism of the occurrence of sarcopenia in the elderly. Sheng Li Xue Bao. (2018) 70:445–54. PMID: 30112570

[113] Zhang YJ Zhao Y Rong JQ Liu KQ Zhan YF Chai YL. A bibliometric analysis of inflammation in sarcopenia from 2007 to 2022. Exp Gerontol. (2023) 183:316. doi: 10.1016/j.exger.2023.112316, PMID: 37862732

[114] Cheng Y Lin SJ Cao ZY Yu RZ Fan YQ Chen J. The role of chronic low-grade inflammation in the development of sarcopenia: advances in molecular mechanisms. Int Immunopharmacol. (2025) 147:4056. doi: 10.1016/j.intimp.2025.114056, PMID: 39799736

[115] Zhang N Zhai LT Wong RMY Cui C Law SW Chow SKH. Harnessing immunomodulation to combat sarcopenia: current insights and possible approaches. Immunity Ageing. (2024) 21:458. doi: 10.1186/s12979-024-00458-9, PMID: 39103919 PMC11299351

[116] Tobias GC Gomes JLP Fernandes LG Voltarelli VA de Almeida NR Jannig PR. Aerobic exercise training mitigates tumor growth and cancer-induced splenomegaly through modulation of non-platelet platelet factor 4 expression. Sci Rep. (2023) 13:21970. doi: 10.1038/s41598-023-47217-2, PMID: 38081853 PMC10713653

[117] Xu HY Brown JL Bhaskaran S Van Remmen H. Reactive oxygen species in the pathogenesis of sarcopenia☆. Free Radic Biol Med. (2025) 227:446–58. doi: 10.1016/j.freeradbiomed.2024.11.046, PMID: 39613046 PMC11816180

[118] Brioche T Lemoine-Morel S. Oxidative stress, sarcopenia, antioxidant strategies and exercise: molecular aspects. Curr Pharm Des. (2016) 22:2664–78. doi: 10.2174/1381612822666160219120531, PMID: 26891808

[119] Chen MM Wang YY Deng SL Lian ZX Yu K. Skeletal muscle oxidative stress and inflammation in aging: focus on antioxidant and anti-inflammatory therapy. Front Cell Dev Biol. (2022) 10:130. doi: 10.3389/fcell.2022.964130, PMID: 36111339 PMC9470179

[120] Scicchitano BM Pelosi L Sica G Musarò A. The physiopathologic role of oxidative stress in skeletal muscle. Mech Ageing Dev. (2018) 170:37–44. doi: 10.1016/j.mad.2017.08.009, PMID: 28851603

[121] Luo XQ Wang J Ju QQ Li TY Bi XL. Molecular mechanisms and potential interventions during aging-associated sarcopenia. Mech Ageing Dev. (2025) 223:2020. doi: 10.1016/j.mad.2024.112020, PMID: 39667622

[122] Ferri E Marzetti E Calvani R Picca A Cesari M Arosio B. Role of age-related mitochondrial dysfunction in sarcopenia. Int J Mol Sci. (2020) 21:236. doi: 10.3390/ijms21155236, PMID: 32718064 PMC7432902

[123] Lo JH Pong KU Yiu T Ong MT Lee WY. Sarcopenia: current treatments and new regenerative therapeutic approaches. J Orthop Transl. (2020) 23:38–52. doi: 10.1016/j.jot.2020.04.002, PMID: 32489859 PMC7256062

[124] Kamarulzaman NT Makpol S. The link between mitochondria and sarcopenia. J Physiol Biochem. (2025) 81:1–20. doi: 10.1007/s13105-024-01062-7, PMID: 39969761 PMC11958477

[125] Leduc-Gaudet J-P Hussain SNA Barreiro E Gouspillou G. Mitochondrial dynamics and Mitophagy in skeletal muscle health and aging. Int J Mol Sci. (2021) 22:179. doi: 10.3390/ijms22158179, PMID: 34360946 PMC8348122

[126] Bernabeu-Wittel M Gomez-Diaz R Gonzalez-Molina A Vidal-Serrano S Diez-Manglano J Salgado F. Oxidative stress, telomere shortening, and apoptosis associated to sarcopenia and frailty in patients with multimorbidity. J Clin Med. (2020) 9:2669. doi: 10.3390/jcm9082669, PMID: 32824789 PMC7464426

[127] Cheema N Herbst A McKenzie D Aiken JM. Apoptosis and necrosis mediate skeletal muscle fiber loss in age-induced mitochondrial enzymatic abnormalities. Aging Cell. (2015) 14:1085–93. doi: 10.1111/acel.12399, PMID: 26365892 PMC4693455

[128] Wang D Song M Shen L-f Han L Zhu P Jia X. Exercise capacity is improved by Levosimendan in heart failure and sarcopenia via alleviation of apoptosis of skeletal muscle. Front Physiol. (2022) 12:895. doi: 10.3389/fphys.2021.786895, PMID: 35126176 PMC8811365

[129] Kim I-Y Park S Jang J Wolfe RR. Understanding muscle protein dynamics: technical considerations for advancing sarcopenia research. Ann Geriatr Med Res. (2020) 24:157–65. doi: 10.4235/agmr.20.0041, PMID: 32752586 PMC7533194

[130] Wu J Ding Pa Wu H Yang P Guo H Tian Y. Sarcopenia: molecular regulatory network for loss of muscle mass and function. Front Nutr. (2023) 10:7200. doi: 10.3389/fnut.2023.1037200, PMID: 36819699 PMC9932270

[131] Cheon Y-H Lee C-H Chung C-H Kim J-Y Lee M-S. Vigeo promotes Myotube differentiation and protects dexamethasone-induced skeletal muscle atrophy via regulating the protein degradation, AKT/mTOR, and AMPK/Sirt-1/PGC1α signaling pathway in vitro and in vivo. Nutrients. (2024) 16:2687. doi: 10.3390/nu16162687, PMID: 39203823 PMC11357481

[132] Yamanashi K Kinugawa S Fukushima A Kakutani N Takada S Obata Y. Branched-chain amino acid supplementation ameliorates angiotensin II-induced skeletal muscle atrophy. Life Sci. (2020) 250:117593. doi: 10.1016/j.lfs.2020.117593, PMID: 32234320

[133] Yoo S-Z No M-H Heo J-W Park D-H Kang J-H Kim SH. Role of exercise in age-related sarcopenia. J Exerc Rehabil. (2018) 14:551–8. doi: 10.12965/jer.1836268.134, PMID: 30276173 PMC6165967

[134] Scudese E Marshall AG Vue Z Exil V Rodriguez BI Demirci M. 3D mitochondrial structure in aging human skeletal muscle: insights into MFN-2-mediated changes. Aging Cell. (2025) 24:e70054. doi: 10.1111/acel.70054, PMID: 40285369 PMC12266761

[135] Aragon AA Tipton KD Schoenfeld BJ. Age-related muscle anabolic resistance: inevitable or preventable? Nutr Rev. (2023) 81:441–54. doi: 10.1093/nutrit/nuac062, PMID: 36018750

[136] Kiseleva OI Arzumanian VA Ikhalaynen YA Kurbatov IY Kryukova PA Poverennaya EV. Multiomics of aging and aging-related diseases. Int J Mol Sci. (2024) 25:671. doi: 10.3390/ijms252413671, PMID: 39769433 PMC11677528

[137] Iwata Y Suzuki N Ohtake H Kamauchi S Hashimoto N Kiyono T. Cancer cachexia causes skeletal muscle damage via transient receptor potential vanilloid 2-independent mechanisms, unlike muscular dystrophy. J Cachexia Sarcopenia Muscle. (2016) 7:366–76. doi: 10.1002/jcsm.12067, PMID: 27239414 PMC4864294

[138] Swiderski K Trieu J Chee A Naim T Brock CJ Baum DM. Altering phosphorylation of dystrophin S3059 to attenuate cancer cachexia. Life Sci. (2025) 362:343. doi: 10.1016/j.lfs.2024.123343, PMID: 39740759

[139] Wang YF Dong ZK An ZY Jin WL. Cancer cachexia: focus on cachexia factors and inter-organ communication. Chin Med J. (2024) 137:44–62. doi: 10.1097/CM9.0000000000002846, PMID: 37968131 PMC10766315

[140] Wang B Thapa S Zhou T Liu H Li L Peng G. Cancer-related fatigue and biochemical parameters among cancer patients with different stages of sarcopenia. Support Care Cancer. (2020) 28:581–8. doi: 10.1007/s00520-019-04717-0, PMID: 31102055

[141] Zhang F-M Wu H-F Shi H-P Yu Z Zhuang C-L. Sarcopenia and malignancies: epidemiology, clinical classification and implications. Ageing Res Rev. (2023) 91:102057. doi: 10.1016/j.arr.2023.102057, PMID: 37666432

[142] He Y Xie W Li H Jin H Zhang Y Li Y. Cellular senescence in sarcopenia: possible mechanisms and therapeutic potential. Front Cell Dev Biol. (2022) 9:88. doi: 10.3389/fcell.2021.793088, PMID: 35083219 PMC8784872

[143] Heitman K Alexander MS Faul C. Skeletal muscle injury in chronic kidney disease-from histologic changes to molecular mechanisms and to novel therapies. Int J Mol Sci. (2024) 25:117. doi: 10.3390/ijms25105117, PMID: 38791164 PMC11121428

[144] Simoes e Silva AC Oliveira EAA Cheung WWW Mak RRH. Redox signaling in chronic kidney disease-associated cachexia. Antioxidants. (2023) 12:945. doi: 10.3390/antiox12040945, PMID: 37107320 PMC10136196

[145] Peng H Cao J Yu RZ Danesh F Wang YL Mitch WE. CKD stimulates muscle protein loss via rho-associated protein kinase 1 activation. J Am Soc Nephrol. (2016) 27:509–19. doi: 10.1681/ASN.2014121208, PMID: 26054539 PMC4731120

[146] Wang K Liu Q Tang M Qi G Qiu C Huang Y. Chronic kidney disease-induced muscle atrophy: molecular mechanisms and promising therapies. Biochem Pharmacol. (2023) 208:115407. doi: 10.1016/j.bcp.2022.115407, PMID: 36596414

[147] Wang S Pan Y Pang Q Zhang A. Irisin ameliorates muscle atrophy by inhibiting the upregulation of the ubiquitin-proteasome system in chronic kidney disease. Calcif Tissue Int. (2024) 115:712–24. doi: 10.1007/s00223-024-01283-4, PMID: 39283327

[148] Wang Y Golledge J. Neuronal nitric oxide synthase and sympathetic nerve activity in neurovascular and metabolic systems. Curr Neurovasc Res. (2013) 10:81–9. doi: 10.2174/156720213804805963, PMID: 23151079

[149] Zhang H Qi GD Wang KX Yang JW Shen YT Yang XM. Oxidative stress: roles in skeletal muscle atrophy. Biochem Pharmacol. (2023) 214:5664. doi: 10.1016/j.bcp.2023.115664, PMID: 37331636

[150] Motoki T Shimizu-Motohashi Y Komaki H Mori-Yoshimura M Oya Y Takeshita E. Treatable renal failure found in non-ambulatory Duchenne muscular dystrophy patients. Neuromuscul Disord. (2015) 25:754–7. doi: 10.1016/j.nmd.2015.07.006, PMID: 26298609

[151] Bergsma A Janssen M Geurts ACH Cup EHC de Groot IJM. Different profiles of upper limb function in four types of neuromuscular disorders. Neuromuscul Disord. (2017) 27:1115–22. doi: 10.1016/j.nmd.2017.09.003, PMID: 29033278

[152] Li J Fredericks M Cannell M Wang K Sako D Maguire MC. ActRIIB:ALK4-fc alleviates muscle dysfunction and comorbidities in murine models of neuromuscular disorders. J Clin Invest. (2021) 131:634. doi: 10.1172/JCI138634, PMID: 33586684 PMC7880416

[153] Nagano K. Change in polygon shapes of atrophic and small angular fibers in disuse muscle atrophy. Muscle Ligaments Tendons J. (2025) 15:66–70. doi: 10.32098/mltj.01.2025.08

[154] Kwon JB Gersbach CA. Directing skeletal myogenic progenitor cell lineage specification with CRISPR/Cas9 transcriptional activators. Mol Ther. (2016) 24:3434. doi: 10.1016/S1525-0016(16)33434-7 PMC7221109 32330446

[155] Ichii S Matsuoka I Okazaki F Shimada Y. Zebrafish models for skeletal muscle senescence: lessons from cell cultures and rodent models. Molecules. (2022) 27:625. doi: 10.3390/molecules27238625, PMID: 36500717 PMC9739860

[156] Kirk B Feehan J Lombardi G Duque G. Muscle, bone, and fat crosstalk: the biological role of Myokines, Osteokines, and Adipokines. Curr Osteoporos Rep. (2020) 18:388–400. doi: 10.1007/s11914-020-00599-y, PMID: 32529456

[157] Nelke C Dziewas R Minnerup J Meuth SG Ruck T. Skeletal muscle as potential central link between sarcopenia and immune senescence. EBioMedicine. (2019) 49:381–8. doi: 10.1016/j.ebiom.2019.10.034, PMID: 31662290 PMC6945275

[158] Wang TT. Searching for the link between inflammaging and sarcopenia. Ageing Res Rev. (2022) 77:1611. doi: 10.1016/j.arr.2022.101611, PMID: 35307560

